# 1997年和2007年湖南地区908例原发性肺癌临床流行病学及病理特征的比较分析

**DOI:** 10.3779/j.issn.1009-3419.2010.04.11

**Published:** 2010-04-20

**Authors:** 芳 李, 瓅 黄, 成平 胡, 其华 顾, 小霞 祝

**Affiliations:** 410007 长沙，中南大学湘雅医院呼吸科 Department of Respiratory Medicine, Xiangya Hospital, Central South University, Changsha 410007, China

**Keywords:** 肺肿瘤, 流行病学, 性别, 吸烟, Lung neoplasms, Epidemiology, Gender, Smoking

## Abstract

**背景与目的:**

肺癌的流行病学特征通常随时间和地区的变化而变化，此研究拟通过比较分析1997年和2007年湖南地区908例肺癌新发病例性别、年龄、城乡来源、吸烟史以及病理类型的差异，以初步了解近年来湖南地区肺癌的流行趋势。

**方法:**

收集湘雅医院1997年和2007年收住的、祖居或长期居住湖南地区的908例初诊肺癌患者的临床资料并进行统计学分析。

**结果:**

与1997年相比，2007年肺癌患者的男女比例由3.8:1降至2.98:1，青年肺癌患者由4.4%升至8.6%（χ^2^=4.465, *P*=0.035），农村肺癌患者由19.9%升至40.1%（χ^2^=30.670, *P* < 0.001），农村肺癌患者中的吸烟者由16.9%升至39.9%（χ^2^= 24.939, *P* < 0.01），少见病理类型肺癌由1.3%升至4.5%（χ^2^= 5.142, *P*=0.023）。

**结论:**

近年来湖南地区女性、青年、农村肺癌患者以及少见病理类型肺癌比例可能有增加趋势，戒烟教育亟待加强。

## Abstract (en)

**Background and objective:**

Epidemiology of lung cancer will be changed along with time and region. The aim of this study is to acknowledge the tendency of primary lung cancer in hunan province in recent years by comparing and analyzing the distribution of gender, age, area, smoking and pathology of patients who were initial diagnosed lung cancer and ancestral or permanent residence of hunan province in 1997 and 2007.

**Methods:**

Clinical data of 908 patients with primary lung cancer hospitalized in Xiangya hospital were collected and evaluated.

**Results:**

Compared patients in 2007 with those in 1997, ratio between male and female dropped from 3.8:1 to 2.98:1, while the proportion of young patients who were under 40 years old raised from 4.4% to 8.6% (χ^2^=4.465, *P*=0.035), patients living in the county raised from 19.9% to 40.1% (χ^2^=30.670, *P* < 0.001), smoking rate of patients from county raised from 16.9% to 39.9% (χ^2^= 24.939, *P* < 0.01). In addition, the proportion of rare histological types of lung cancer were also increased from 1.3% to 4.5% (χ^2^= 5.142, *P*=0.023).

**Conclusion:**

Female patients, young patients, rural patients and rare histological types of lung cancer may have a tendency of increase in hunan province in recent years, whereas smoking cessation education should be strengthened.

肺癌是一种严重危害人类健康的恶性肿瘤，近年来其发病率和死亡率在世界许多国家和地区都明显上升，但流行趋势却较前有所变化并且具有明显的地区差异^[1, 2]^。因此，本文拟通过对中南大学湘雅医院1997年和2007年收住的、祖居或长期居住湖南地区的初诊肺癌患者性别、年龄、城乡来源、吸烟史以及病理类型的比较分析，旨在初步了解近年来湖南地区原发性肺癌的临床流行病学特点以及病理类型的发展变化。

## 1 对象和方法

### 1.1 研究对象

通过病案信息科收集1997年和2007年湘雅医院收治的肺癌新发病例908例，所选病例均祖居或长期居住湖南地区，均通过痰液、胸水、支气管镜、肺穿刺及手术等方法获得病理学依据，并通过统一的调查表获得患者详细的个人信息和临床资料，包括性别、年龄、城乡来源、吸烟史以及病理类型。

### 1.2 统计学方法

Excel软件录入数据，双人校对及应用SPSS 15.0软件逻辑纠错后进行统计学分析，包括对1997年和2007年的肺癌患者性别、年龄、城乡分布、吸烟史以及病例类型的描述性分析和比较分析，计数资料采用χ^2^检验或*Fisher*确切概率法，计量资料采用*t*检验，*t*检验前先行方差齐性检验，*P* < 0.05为差异具有统计学意义。

## 2 结果

### 2.1 肺癌患者的一般情况

如表 1所示，1997年肺癌患者共计231例（25.4%），男女比例为3.8:1，发病年龄最小21岁，最大78岁，平均发病年龄55.58岁，其中吸烟患者154例（66.6%），包括城市128例（83.1%）、农村26例（16.9%）。2007年共计677例，男女比例为2.98:1，发病年龄最小3岁，最大82岁，平均年龄56.03岁，其中吸烟患者429例（63.4%），包括城市258例（60.1%）、农村171例（39.9%）。1997年和2007年肺癌患者均主要集中在40岁以上的中年（40岁-60岁）、老年（> 60岁）肺癌组（95.6%, 91.4%），并且大部分居住在城市（80.1%, 59.9%），病理类型均以鳞癌为主（46.3%, 40.0%）。两个时间段的肺癌患者在性别构成、平均发病年龄以及吸烟率等方面均无统计学差异（*P* > 0.05）。但2007年女性肺癌患者增加254%，比男性患者17 7%的增加趋势更为明显。青年肺癌患者（由4.4%升至8.6%, χ^2^= 4.465, *P*= 0.035）、农村肺癌患者（19.9%升至4 0.1%, χ^2^=30.670, *P* < 0.001）、农村肺癌患者中的吸烟者（16.9%升至39.9%, χ^2^= 24.939, *P* < 0.01）明显增加。2007年少见病理类型肺癌尤其是除腺鳞癌以外的混合癌的比例亦明显增多（*P*=0.016），而腺癌比例无明显变化（*P*=0.957）。

**1:** 908例肺癌患者的一般情况 Characteristics of 908 patients with lung cancer

Item	1997 (n=231)	2007 (*n*=677)	χ^2^	*P*
Gender			1.771	0.183
Male	183	507		
Female	48	170		
Age				
< 40	10	58	4.465	0.035
40-60	127	367	0.041	0.840
> 60	94	252	0.879	0.348
Area			30.670	0.999
City	185	406		
County	46	271		
Smorking			0.816	0.366
Yes	154	429		
No	79	248		
Pathology				
Squamous cell carcinoma	107	271	2.805	0.094
Adenocarcinoma	82	239	0.002	0.957
Adeno squamous cell carcinoma	10	25	0.188	0.664
Small cell lung cancer	24	89	1.201	0.273
Bronchiole-alveolar carcinoma	5	22	2.839	0.089
Else	3	31	5.142	0.023
Carcinosarcoma^*^	0	4		0.577
Large cell lung Cancer^*^	0	1		0.999
Pulmonary blastoma^*^	0	1		0.999
Carcinoid^*^	2	2		0.269
Shuttle cell carcinoma^*^	0	1		0.999
Mucoepidermoid carcinoma^*^	1	7		0.687
Mixed-carcinoma^*^	0	15		0.016
^*^The number is too few，so we use *Fisher’s* exact test，not χ^2^ value.				

### 2.2 肺癌病理类型与性别

如表 2所示，2007年与1997年男女肺癌患者各自的肺癌病理类型分布均无统计学差异（χ^2^=8.879, *P*=0.114; χ^2^=2.405, *P*=0.791），其中男性肺癌患者均以鳞癌为主（51.4%, 46.7%），女性患者均以腺癌为主（47.9%, 51.2%）。

**2:** 肺癌各病理类型的性别分布 Distribution of pathology in different gender

Pathology	1997			2007	
	Male	Female		Male	Female
Squamous cell carcinoma	94	13		237	34
Adenocarcinoma	59	23		152	87
Adeno squamous cell carcinoma	8	2		18	7
Small cell lung cancer	20	4		70	19
Bronchiole-alveolar carcinoma	0	5		8	14
Else	2	1		22	9
Total	183	48		507	170

### 2.3 肺癌病理类型与年龄

如表 3所示，1997年与2007年青年肺癌患者病理类型均以腺癌为主（50%, 50%），而中老年肺癌患者则以鳞癌为主（42.5%, 55.3%; 40.1%, 44.8%）。两个时间段相比，除中年肺癌组少见病理类型肺癌明显增加外（χ^2^=12.270, *P*=0.031），2007年青年肺癌组及老年肺癌组各自的肺癌病理类型构成均无统计学差异（χ^2^=5.274, *P*=0.383; χ^2^= 4.119, *P*=0.532）。

**3:** 肺癌各病理类型在不同年龄段的分布 Distribution of pathology in different ages

Pathology	1997				2007		
	< 40	40-60	> 60		< 40	40-60	> 60
Squamous cell carcinoma	1	54	52		11	147	113
Adenocarcinoma	5	50	27		29	124	86
Adeno squamous cell carcinoma	1	5	4		0	15	10
Small cell lung cancer	1	15	8		10	48	31
Bronchiole-alveolar carcinoma	0	3	2		1	16	5
Else	2	0	1		7	17	7
Total	10	127	94		58	367	252

### 2.4 肺癌病理类型与地区

如表 4所示，两个时间段城市肺癌患者病理类型均以鳞癌为主（54.3%, 42.3%），但2007年肺鳞癌比例较1997年下降（χ^2^=8.559, *P*=0.003），而其他一些少见病例类型肺癌增加明显（χ^2^=8.566, *P*=0.003）；农村肺癌患者的病理构成虽然无明显差异（χ^2^=3.026, *P*=0.696），但2007年农村肺癌患者病理类型由以腺癌为主（40.6%）转变为以鳞癌为主（53.1%）。

**4:** 肺癌各病理类型的城乡分布 Distribution of pathology between city and county

Pathology	1997			2007	
	City	County		City	County
Squamous cell carcinoma	91	16		148	123
Adenocarcinoma	63	19		146	93
Adeno squamous cell carcinoma	8	2		18	7
Small cell lung cancer	19	5		55	34
Bronchiole-alveolar carcinoma	3	2		16	6
Else	1	2		23	8
Total	185	46		406	271

### 2.5 肺癌病理类型与吸烟

如表 5所示，2007年与1997年肺癌患者中吸烟者与非吸烟者各自病理类型分布均无明显变化（χ^2^=9.886, *P*=0.079;χ^2^=2.954, *P*=0.707），其中吸烟者均以鳞癌为主（57.8%, 51.9%），而非吸烟者均以腺癌为主（52.5%, 52.6%）。

**5:** 肺癌病理类型与吸烟的关系 Relationship between pathology and smoking

Pathology	1997			2007	
	Smoking	Non-smoking		Smoking	Non-smoking
Squamous cell carcinoma	89	18		223	48
Adenocarcinoma	40	42		108	131
Adeno squamous cell carcinoma	6	4		15	10
Small cell lung cancer	18	6		61	28
Bronchiole-alveolar carcinoma	0	5		6	16
Else	1	2		16	15
Total	154	77		429	248

## 3 讨论

肺癌是当今世界最常见的恶性肿瘤，2007年全世界恶性肿瘤新发病例超过1 200万例，死亡760万例，其中肺癌新发病例150万例，占12%，死亡135万例，成为全世界发病率和死亡率最高的癌症。几乎在所有国家肺癌男性发病率和死亡率均明显高于女性^[2]^，但近年来我国上海及某些欧美国家出现女性肺癌较男性肺癌上升增快的趋势^[3, 4]^。通过对该院两个时间段肺癌患者的性别统计，结果显示1997年该院肺癌患者男女比例为3.8:1，2007年则降至2.98:1，虽然两者无统计学差异（*P*=0.183），但2007年女性肺癌患者增加254%，同比男性患者增加177%，提示该地区女性肺癌患者增加速度亦可能较男性明显。虽然女性肺癌的发病机制目前仍未完全阐明，但近年来认为女性除在雌激素和雌激素受体水平上与男性不同外，还在代谢酶基因如细胞色素p450 1A1酶（CYP1A1酶）、DNA修复基因如*XPD*基因Asp312Asn和Lys751Gln多态性、胃泌素多肽表达及易发生基因变异如P53等方面与男性存在差异，并由此导致女性对室外烟草烟雾中尼古丁、空气中排放的多环芳烃等以及室内燃煤、油烟污染等致癌物的基因修复能力较男性减弱，因而随着环境污染的加剧，女性罹患肺癌尤其是腺癌的风险较男性明显增加^[5-8]^。

肺癌多见于中老年患者，通过对该院2007年和1997年肺癌患者发病年龄的比较分析发现，尽管两者的平均发病年龄无统计学差异（*P*=0.592），但2007年青年肺癌患者明显增加（*P*=0.035）。与此同时，两个时间段青年肺癌患者病例类型均以腺癌为主（50%, 50%），从而预示其病情进展快、转移早、治疗效果差。然而，由于目前医务人员以及患者对青年肺癌缺乏应有的警惕性、对其特殊临床表现认识不足以及常规X线等无创检查存在较高误诊率等原因，导致青年肺癌的误诊率高达85.7%^[9]^。宋敏等^[10]^对该院呼吸科1996年1月-2006年12月10年间经支气管镜活检、最终病理证实的469名青年肺癌进行统计，发现支气管镜诊断青年肺癌的符合率达到82.9%，远高于报道的胸部CT的73%的符合率^[11]^。因此，对于临床可疑肺癌的青年患者尤其是青少年患者（< 25岁），应及时行支气管镜检查明确病因，避免误诊、漏诊，贻误治疗^[12]^。

与我国肺癌流行特征相似^[13]^，该院两个时间段均以居住在城市的肺癌患者为主，但2007年居住在农村的肺癌患者明显增加（*P* < 0.001）。除此之外，在肺癌病理类型的构成上，2007年居住在农村的肺癌患者也从以腺癌为主转变为以鳞癌为主。造成农村肺癌患者增多的原因有很多，其中一方面可能是农村居民近年来多用煤替代生物燃料（庄稼秸杆、木材、木棍和树枝）来取暖和做饭，目前虽无报道显示煤烟暴露与肺癌有明显关系，但煤烟中的多环芳烃和苯并芘已被证实与肺癌的发病密切相关^[14]^；而另一方面则可能是由于收入的增加以及新型农村合作医疗制度的出台，使农村居民有能力到省级医院就诊，从而在一定程度上也增加了农村肺癌患者的比例。

吸烟目前仍被认为是肺癌的主要危险因素，且与鳞癌的发生密切相关^[15]^。该院两个时间段肺癌患者中的吸烟者分别占同期肺癌患者的66.6%和63.4%，病理类型均以鳞癌为主，而非吸烟者则均以腺癌为主。与1997年相比，虽然2007年肺癌患者中的吸烟者比例无明显变化（*P*=0.366），但农村肺癌患者中的吸烟比例明显增加（*P* < 0.01），并可能由此导致了上述农村肺癌患者由以腺癌为主向以鳞癌为主的转变。因此，应进一步加大控烟教育的力度和范围，提高民众尤其农村民众对吸烟有害健康的认识，降低肺癌的发病率和死亡率。

关于肺癌各种病理类型的分布，有报道^[1]^指出近年来肺腺癌发病率的速度在明显上升。虽然此分析未发现1997年与2007年该院肺癌患者中腺癌构成比存在明显差异（*P*=0.957），但2007年一些少见病理类型肺癌尤其是少见的混合型肺癌的诊断明显增多，因其没有与其它病理类型肺癌相区别的特征性X线表现，又兼具各种成分癌的特性，因此当患者尤其是居住在城市的中年肺癌患者病理诊断为单一成分癌，而手术、放疗及化疗不能达到预期效果时，应考虑混合癌存在的可能。

综上所述，近年来湖南地区女性肺癌患者、青年肺癌患者、农村肺癌患者可能有增加的趋势，临床上应警惕少见病理类型肺癌，并进一步加大农村及中小城市居民的控烟教育。
